# Developing a Meaningful Garden Space in a Care Home with Staff and Family Members: A Qualitative Study

**DOI:** 10.3390/ijerph19127025

**Published:** 2022-06-08

**Authors:** Clarissa Giebel, Bram de Boer, Mark Gabbay, Caroline Watkins, Neil Wilson, Hilary Tetlow, Hilde Verbeek

**Affiliations:** 1 Department of Primary Care & Mental Health, University of Liverpool, Liverpool L69 3GL, UK; mbg@liverpool.ac.uk; 2 National Institute for Health Research Applied Research Collaboration North West Coast, Liverpool L69 3GL, UK; clwatkins@uclan.ac.uk; 3 Care and Public Health Research Institute, Maastricht University, 6229 ET Maastricht, The Netherlands; b.deboer@maastrichtuniversity.nl (B.d.B.); h.verbeek@maastrichtuniversity.nl (H.V.); 4 Applied Sciences for Health Hub, UCLAN, Preston PR1 2HE, UK; nwilson9@uclan.ac.uk; 5 SURF Liverpool, Liverpool L8 7SS, UK; htetlow@btinternet.com

**Keywords:** care homes, garden, meaningful activities, co-production

## Abstract

**Background:** Care home residents are often passive and lack active engagement in meaningful activities. The aim of this qualitative study was to co-develop a plan for a meaningful garden space in an urban care home in the north of England, to inform the subsequent building of such a garden space on the care home premises. **Methods:** Members of staff participated in focus groups conducted at the care home. Family carers were interviewed by telephone. Both focus groups and interviews were audio-recorded, with transcripts analysed independently using thematic analysis by two researchers, and consensus achieved on final themes. **Findings:** Two focus groups with staff (n = 17) and seven interviews with family carers were held. Thematic analysis generated seven key themes for the garden about its meaning and environmental features: (1) Current opinions on physical environment; (2) access; (3) adaptation to the environment; (4) staffing; (5) socialising; (6) sensory features; and (7) active meaningful participation. The garden needed to be accessible to residents in wheelchairs, and in all weathers and seasonal conditions, as well as being adapted to the needs of people living with dementia. Areas for social activities, such as picnics, and intergenerational activities, as well as private spaces, were recommended. Throughout the garden, sensory features were suggested, incorporating the use of vision, smell, touch, and sound, such as through water features. Moreover, it was recommended that residents should be able to contribute to the delivery of the activities themselves, including through a café and a vegetable garden. **Conclusions:** Family carers and staff considered that the garden would benefit from an intensive update to meet the needs of residents. This study therefore has practical implications for care home design, which are of even greater importance since the pandemic, as outdoor spaces were considered safer for care home visiting.

## 1. Introduction

A quarter of a million people aged 65 and above are living in institutional long-term care (LTC) facilities, including care homes and nursing homes, in England [1]. In the UK, there are approximately 17,600 care homes caring for vulnerable people with long-term care needs ([2]). Many of those living in a care home have dementia, and therefore have specific needs such as challenges with everyday functioning [3], cognitive problems [4] and behavioural difficulties [5]. With an estimated 55 million people living with dementia worldwide [6], there is a growing need for adequate LTC facilities.

With many care home residents adopting a passive approach in their day-to-day lives, and care home staff usually performing activities for the residents as opposed to doing activities with them [7,8], the importance of the physical environment for the well-being of people with dementia is well recognised. The built environment can support people with dementia to attain their full potential by positively influencing their autonomy, support their quality of life and well-being and attain the best possible potential for independence [9,10]. Literature reviews confirm the importance of the environment for people with dementia (e.g., sunlight, sounds, view, spatial layout, nature, orientation, music, privacy, autonomy, windows, comfort, facilities, staff, group size, non-institutional character, and domesticity) [11,12].

The substantial evidence of the role of the physical environment for people with dementia is especially relevant to the care home and nursing home sector. There is increasing interest in the physical design of the care or nursing home environment and how this supports person-centred care [9,13,14]. For instance, a home-like environment can positively influence residents’ daily activities and social interactions [15] and being outdoors is associated with having positive mood [16]. Significant benefits from being outdoors and using garden spaces have been reported for people living with dementia [17].

The developing of garden spaces in care homes is starting to be explored. Understanding the important features of such a garden space relevant to the intended users would be important. A recent conceptual framework for garden spaces for frail elderly, constructed based on a literature review, included different dimensions and aspects, such as social uses, attractiveness of the outdoor space, secure environments, accessibility, composition, and climate conditions [18]. The social uses of the outdoor space were also highlighted as an important feature of a beneficial garden space, and are all the more important during the COVID-19 pandemic, where many visits occur outside to reduce the spread of the virus. Whilst the authors have generated these features via a literature review and consultation with relevant stakeholders, this was an evidence-based approach to the features, as opposed to a bottom-up approach, with ideas generated directly from those working in care homes or living in/visiting a relative in a care home. Improved well-being and higher levels of social interaction have been achieved when care home residents engage with garden outdoor spaces and felt connected to them [19]. It is important to address and implement these features and elements in order to engage care home residents.

The need for suitable outdoor or garden spaces for family carers to visit residents has been heightened because of COVID-19, to limit the spread of the virus to the vulnerable care home population. Meeting family and friends outdoors in a socially distanced way is considered safer than meeting indoors. Whilst a trial of gradual re-opening of 26 care homes in the Netherlands has proven to benefit the residents’ well-being [20], even in the UK, care home residents are still unable to fully return to pre-pandemic social activity levels [21]. The importance of inviting and useful outdoor and garden spaces has been highlighted since the COVID-19 pandemic, as a means of meeting and socialising at distance with good airflow to reduce transmission risk.

Staff and family carers have rarely been reported to be involved in the development and design of a care home [22] and garden spaces [18,19]. Instead, it is more common that gardens are designed by managers, owners and architects with limited or no consultation, which risks failing to adequately address the needs and opinions of residents and family carers [23]. Co-production [24] can support the meaningful development of a garden space. This can be achieved by discussing with relevant stakeholders (in this case, care home staff and family carers) their opinions on how the garden should be built and what to take into consideration to increase usage. By identifying the wishes and ideas of those who will be using and providing access to the garden, the expectation is that the new garden that is implemented would be more likely to be relevant and meaningful. Therefore, the aim of this study was to involve family members and care home staff in developing a meaningful garden space in a care home, which is all the more important with COVID-19 having placed severe restrictions on visitation.

## 2. Methods

### 2.1. Participants and Recruitment

We were invited by a care home organisation to run the co-production in developing a garden space further. We obtained ethical approval from the University of Liverpool [Ref: 5198]. This research was conducted in a care home that had 86 residents and a special dementia ward, located at the suburban outskirts of a city in the northwest of England. Participants were recruited via convenience sampling. Care home staff were eligible if they worked in a caring capacity at the care home (rather than administration, cleaning or maintenance). Family carers were eligible if their relative resided in the care home. All participants had to be aged 18 or above. Whilst it was attempted to interview people with dementia, upon Mental Capacity assessment, all residents appeared to lack the capacity to consent, and were thus excluded as potential participants.

Care home staff were approached by one of the research team members, provided with a study information sheet, and were given the opportunity to discuss the project with the research team member. If people were interested in taking part, they were asked to inform the care home manager or a member of staff, or inform the research team directly. Information about the study was advertised and shared within the care home by the manager, so that every care home staff member was aware and could express an interest. Care home staff took part in focus groups at the care home, with written informed consent obtained prior to the focus group. Family carers who were regularly visiting their relatives were contacted via phone by the care home to enquire about their interest in the study. If interested, their contact details were forwarded to the lead researcher who conducted the interviews, after explaining the study and confirming consent.

### 2.2. Procedure

Focus groups were conducted in a quiet room in the care home. Participants were asked about what elements would be considered useful in the garden for the residents to meaningfully engage, and whether the garden needs to be adapted to enable easy access to all residents. Staff were also asked about any potential barriers of using the garden. Focus groups lasted up to 30 min. Additional notes were taken after one of the focus group finished, as staff continued to discuss the topic after the audio recorder was switched off.

Due to the COVID-19 pandemic, interviews with family members were conducted over the phone, with informed verbal consent being taken at the beginning of the telephone interview and audio-recorded. Carers were asked about whether their relative had been using a garden before entering the care home. Interviews lasted up to 15–20 min.

All focus groups and interviews were conducted by the lead researcher (CG). Audio-recordings were subsequently transcribed and anonymised.

### 2.3. Data Analysis

Focus group and interview data were analysed using inductive thematic analysis following the six steps outlined by Braun and Clarke [25], and transcripts were coded manually for themes by two researchers trained in qualitative analysis (CG, BdB). Analysis was carried out in several steps. Both researchers first independently read all transcripts and interviewer notes to familiarise themselves with the data. Transcripts were then coded, and subsequently categorised by combining codes that were similar, creating a list of themes. Both researchers discussed these generated codes and themes, reaching consensus on a final number of themes.

## 3. Results

A total of 17 members of staff took part in two focus groups. Most staff were female (82%) and had worked, on average, for 10 (+/−8) (0.1–20) years at the care home as non-clinical staff members. Seven family carers were interviewed via telephone. Family carers were mostly female (71%) and caring for their parent (85.7%).

### 3.1. Qualitative Findings

Thematic analysis identified seven themes: (1) Current opinion on physical environment; (2) access; (3) adaptation to the environment; (4) staffing; (5) socialising; (6) sensory features; and (7) active meaningful participation. The included quotes are illustrative of the themes and do not represent the entirety of the quotes available.

**THEME 1: Current opinion on physical environment**

### 3.2. Outdoors

Many carers noted that their relatives were not currently using the garden, and that on family visits to the care home, they were reluctant to use the garden with their relatives. Some relatives expressed views about social spaces for people to sit outdoors and garden features to look at, and were discontent with how the garden looked, as it was not well designed and lacked innovative features to be inviting for spending time in. Moreover, some carers were not sure that they were allowed to access the garden.

*“I’ve never been out in the garden there at all. I didn’t really know whether we were allowed to go to be quite honest.”*

***Female carer (daughter), ID07***

*“Outside of his room there’s a patch where there is grass, there should be flowers or something and it’s just mud”*

***Female carer (daughter), ID02***

*“At the moment it’s just sort of, you step out and you sit down at the table and that’s as far as you get and there’s not much growing there to see”*

***Female carer (daughter), ID01***

*“She goes for a walk around there to exercise, she can’t get out but she likes the garden she worked with one of the staff there, her and one of the staff do all the flower baskets and I bring flowers so they can plant them.”*

***Male carer (son), ID04***

### 3.3. Indoors

There were some negative comments about the interior by one family carer in particular, as well as by staff after one focus group had finished. Staff appeared to be more comfortable sharing such comments when they were not recorded. Participants were raising concerns as to why the garden was being adapted and improved, when residents spend most of their time indoors.

*“They should be spending money inside the home basically rather than outside because a lot of the residents they feel the cold a lot they don’t really want to go outside or if they do they’ve got to be wrapped up and then no sooner are they out there they’re like wanting to go back in because they’re cold. But inside the home, everything just a block colour, there’s no bright colours in there.”*

***Female carer (daughter), ID03***

**THEME 2: Access**

To make the outdoor space easily accessible to all residents, several adaptations were suggested. Many residents are wheelchair users, so a wide path to the garden was recommended, as well as general adaptations to enable wheelchair users take part in activities, such as raised flower beds and vegetable garden boxes, as well as tables of suitable height.

*“The easier it is for the staff to manoeuvre round with wheelchairs the more people will take them out”*

***Staff focus group 1***

*“If you did things like growing your own vegetables, they need to be in raised beds so that everybody can take part”*

***Staff focus group 2***

*“She’s not so steady on her pins so their ground would have to be fairly even you know I wouldn’t want her to trip and fall”*

***Female carer (daughter), ID07***

Generally, many family carers were highlighting that there was no clear path from individual rooms leading to the main garden area, but just grass to walk on. This can be particularly difficult for people in a wheelchair or with a walking frame. People living with dementia can become particularly easily disorientated, so that paths should be easy to follow and leading back to where they started. One family carer also stated that there should be better signage to use the garden, as they were not aware that they were allowed to access the garden when visiting their relative.

*“If there was a kind of path or a trail you know that you’d go along at the moment it seems like it’s, where my mum is, it’s just outside the patio door kind of thing and then it kind of stops.”*

***Female carer (daughter), ID01***

*“There isn’t an actual pathway its grass from his room but its grass where you can push the wheelchair, its ok so there needs to be some sort of access from their rooms.”*

***Female carer (daughter), ID02***

*“It’s got to help the residents and they’ve got to be able they’ve got to be able to feel safe and happy. So for the residents to get out the home while family aren’t there, there’s, I think where the dining area is there’s a couple of French doors where you can just about get a wheelchair out of them to be honest […] it’s not wheelchair friendly.”*

***Female carer (daughter), ID03***

**THEME 3: Adaptations to variable seasons and weather**

To ensure that the garden can be used as often as possible, adaptations for different weather and seasons were suggested. A shelter from the sun and the rain should be part of the outdoor space, whilst staff were also concerned about garden maintenance during colder months. Staff were concerned that the garden would not be looked after by a gardener properly during wintertime, which would hinder staff from going outdoors with residents.

*“They need shelter; they go out more if it was a sheltered area”*

***Staff focus group 2***

*“Maybe some heaters lamps so it was warm out there in the winter”*

***Female carer (daughter), ID05***

**THEME 4: Staffing**

Staffing issues were only raised by care home staff. They raised the importance of having sufficient staff to allow residents to use the garden space. If many residents wanted to use the new garden simultaneously, they suggested a rota for different groups of residents going outdoors. Staffing issues were also raised for maintaining certain recommended meaningful activities (see also Theme 7), such as a kiosk.

*“We’d have to have extra staff on to take them out ‘cause then you’re leaving others short”*

***Staff focus group 2***

*“Where at the moment once they’re, unless somebody—takes them out in a wheelchair or walks them round from the actual unit themselves inside, from all of their windows there’s nothing really nice to look at basically”*

***Female carer (daughter), ID03***

**THEME 5: Socialising**

Having spaces to socialise in the garden were considered very important by staff and family members. This involved a range of different socialising types and settings, including having picnic spaces and seating areas to spend time outdoors with family members who come to visit.

*“I’ve seen in other homes that although they do one area which is what you would class as communal seating so you can sit in groups and all take part and join in, they also did built up beds and they put the seating there so that if families wanted to take their relatives, they can sit on their own if they wish to”*

***Staff focus group 2***

*“It could do I suppose with another bench and seats and that maybe around the corner on the grass when they are you’ve only got the one bench and one table and bench there you know you’ve room to sit I think five or six. Another one around the corner where the grass is would be nice.”*

***Male carer (son), ID04***

Intergenerational activities have come out as an important social element as part of the outdoor space and its activities mostly by staff, as family members might bring their children but not necessarily think that this is something special. This also involved having enough space for grandchildren to play, and, for children from nearby schools to come visit in order to facilitate play and engagement with the residents.

*“Just having a few games outside what the kids can play with the residents and the residents can then obviously engage with the kids”*

***Staff focus group 1***

While social spaces were considered important, staff also raised the value of having spaces for privacy. Residents might not always wish to socially engage with others, be they family members or fellow residents, and at times also want to have the opportunity to enjoy the garden space by themselves.

*“Sometimes they just want to sit nice and quiet to have their own little space.”*

***Staff focus group 2***

**THEME 6: Sensory features**

Implementing various sensory features came out as a strong theme across the staff focus groups and family carer interviews. Staff and family carers generated various ideas on how to stimulate care home residents by means of vision, smell, sound, and touch. This included hanging up wind chimes, creating water features, different colours across the garden, boxes of sand with pebbles to touch different textures, and various flowers to look at and smell. Having pets as part of the outdoor space was also suggested by various staff members, ranging from having ducks to having regular pet therapy at the home.

*“It would be nice to have smells as you’re going along this pathway to get to this meaningful garden […], colours especially for people that are living with dementia”*

***Staff focus group 2***

*“Bird tables you know attract the wildlife squirrels maybe I’d say that they’re you know erm something to keep their attention span going”*

***Female carer (daughter), ID05***

*“Some therapy things you know like all bright colours and something that just makes it look happy”*

***Female carer (niece in law), ID06***

*“Telephone boxes, gardens, shop fronts you know just something basically because you’ve got to think with dementia people they like lots of colour and lot of vibrant colour and difficult to create that using natural plants because of whatever time of year it is. It would be nice to have loads of natural plants with lots of natural colour and what have you.”*

***Female carer (daughter), ID03***

Installing bird tables and enabling residents to feed the birds also came up often by members of staff and family carers. This would not only be an activity, but allow residents to receive visual and auditory stimulation also, in line with the other recommended sensory features.

*“Bird tables and things like that just so the birds all come down and something that the residents could do is feed the birds”*

***Female carer (niece-in-law), ID06***

**THEME 7: Active meaningful participation**

In addition to socialising in the garden space as a key positive feature (see Theme 5), staff across both focus groups independently brought up the idea of building a café outdoors. The café could be open at certain times during the week and days, and would allow care home residents to purchase something with their own money. Staff suggested that residents were keen on purchasing items with their own money, which is not possible in the normal care home setting. Residents were also suggested to be involved in managing the café, jointly with staff, to give them a meaningful activity to get involved with.

*“They can actually purchase something because a lot of residents want to purchase something, they want to go somewhere to actually purchase the drinks […] themselves”*

***Staff focus group 2***

Other suggested activities which could be done in the garden involved moving activities such as crafts organised by the care home’s activity coordinator outdoors, so that residents could enjoy a different environment and engage in the social activity outside. Particularly for residents who had been keen gardeners before entering the care home, creating a vegetable garden and involving the residents in maintaining the garden was suggested by staff and family carers.

*“It would be nice to have an area outside where you know a communal area where maybe if as and when the activity coordinators are out there, you know maybe they could do little crafts or you know a nice big table where people could sit around it and you know she could do amenities with them, little activities”*

***Female carer (daughter), ID03***

*“He likes doing gardening so he probably would enjoy things like doing the vegetables. If someone’s come in to visit him he wouldn’t do anything with you”*

***Female carer (niece in law), ID06***

*“I don’t think my mum’s that sort of like way inclined but she could very well be. But I can see that there must be other keen gardeners in the unit which whom I’m sure would absolutely love to be able to get their hands dirty again. There must be a lot of knowledge there that perhaps you know from people who enjoyed their gardens you know to and to give them the opportunity to have green fingers again and potter in a space outside I’m sure would benefit them greatly.”*

***Female carer (daughter), ID07***

## 4. Discussion

This study provides some of the first evidence on co-producing a meaningful outdoor space for care home residents. This exploratory study showed that both staff and family members raised various design and practical issues of the garden, including sensory features such as water fountains and wind chimes, physical adaptations to allow residents in wheelchairs to access the garden easily as well as orientation features for those residents with cognitive impairment, as well as adaptations to use throughout any weather. The garden should also involve meaningful participation and opportunities for socialising, and participants highlighted different organisational and environmental barriers that might hinder the use of the garden space.

To date, there has been limited evidence on co-producing care home elements, whether inside or outside on the premises. Morgan-Brown and colleagues [26] co-designed and implemented an indoor adaptation to a care home and measured its effects, showing that compared to pre-implementation, residents were more engaged with the adapted living space afterwards. In this study, family carers and staff both mentioned that the interior of the care home should be focused on first, as residents currently spend most of their time indoors. Family carers also raised general concerns about the existing outdoor area, which they suggested acted as a barrier for family carers and residents, stopping them from accessing the garden, and from wanting to access the garden in the first place. One carer in particular expressed dissatisfaction initially with the current indoor and outdoor design, but then actively engaged throughout the interview and provided constructive suggestions on how the outdoor space could be designed.

Even with an ideal garden in place, this may not necessarily lead to increased usage. There needs to be a general perception that it is safe and encouraged to use the garden, which is unlikely to be achieved by only putting up a sign, as suggested by one carer. Using the garden and holding activities outside also needs to be a part of the normal working routine for care home staff and thereby expanding the area of care provision, so that usage is not only dependant on family carers’ perceptions. Considering that natural leisure spaces in care homes are important for residents [16,27], adapting the garden based on this co-production study is important to raise the potential usage of the outdoor space for residents and family carers.

Recommended design features included sensory features, corroborating the benefits of multi-sensory stimulation in people living with dementia [28,29], and enabling socialising spaces for residents, amongst themselves, alone, and with staff and family members. Social isolation and loneliness are a big concern for care home residents [30], with residents often not interacting with one another or not being provided with activities or opportunities to engage socially [31]. However, socialising with other residents and family members is important to maintain a good quality of life [32]. This could be easily encouraged by providing more seating areas in the garden space, as well as individual benches outside each resident’s patio doors, where they are based on the ground floor and have their own access to the garden. Inviting school or kindergarten children over was also suggested by participants, the benefits of which are strongly supported by evidence. Intergenerational activities and social engagement are found to be very beneficial to the well-being of people living with dementia and older adults in general [33,34]. To enable intergenerational activities, some successful elements include a buddy system to build relationships, reminiscence programmes, as well as dementia education for the children prior to the activities [34]. These recommendations made by staff and family carers are thus relatively easy to implement, and should provide a better quality of life for the residents.

This study is subject to some limitations. We only interviewed care home staff and family carers about their views on how the garden should be built. Whilst we tried to talk to residents, upon assessing their mental capacity, they were considered lacking capacity to consent and thus were unable to take part in the study. This limitation relates to the ethical constraints for informed consent to participate in research, rather than the potential benefits of consulting residents in such settings about environmental developments and improvements. However, by talking to their closest kin, many of whom usually visited their relative in the care home several times a week pre-COVID-19, we captured the views of those who know the residents best, whilst care home staff spends a great deal of time with the residents and can judge how care can be delivered safely outside. Moreover, for gatekeeping purposes and data protection, family carers were first approached by the care home to explore whether they would be interested in participation. This may have led to some bias in who the care home contacts; however, we captured both positive and negative experiences of the garden space, which highlights that the family carer sample was not biased towards one opinion. The garden design was strongly influenced by the consultation which took on board all of the feedback. Whilst these findings are not generalisable, as only staff and family members from one care home were involved in the coproduction of this specific garden, this study provides novel guidance on how to approach building an element of a physical environment that is highly important to the well-being of care home residents. The pandemic may well influence the future design of communal areas to facilitate visits that are adaptable to infection-control and prevention initiatives, but this data collection pre-dated these new realities.

## 5. Conclusions

This appears to be one of the first studies to report on the co-production of developing a meaningful outdoor space in a care home. In light of the COVID-19 pandemic, during which care home residents in many countries have not been allowed to see family members and friends, having an accessible and engaging garden is even more important. Findings from this study will shape the design of the garden to be built at this care home, and also provide guidance for future implementation work into co-producing physical environments with the aim of benefitting care home residents.

## Data Availability

The data presented in this study are available on reasonable request from the corresponding author. The data are not publicly available due to ethical restrictions.
